# Recent Methods for Purification and Structure Determination of Oligonucleotides

**DOI:** 10.3390/ijms17122134

**Published:** 2016-12-18

**Authors:** Qiulong Zhang, Huanhuan Lv, Lili Wang, Man Chen, Fangfei Li, Chao Liang, Yuanyuan Yu, Feng Jiang, Aiping Lu, Ge Zhang

**Affiliations:** 1 Institute of Integrated Bioinformedicine and Translational Science, School of Chinese Medicine, Hong Kong Baptist University (HKBU), Hong Kong, China; qlzhang12@163.com (Q.Z.); lvhuan1988@aliyun.com (H.L.); wanglili9413@163.com (L.W.); chen131195@163.com (M.C.); fayebalaba@live.com (F.L.); liangchao512@163.com (C.L.); yu.yy01@hotmail.com (Y.Y.); 2 The State Key Laboratory Base of Novel Functional Materials and Preparation Science, Faculty of Materials Science and Chemical Engineering, Ningbo University, Ningbo 315211, China; 3 Institute of Precision Medicine and Innovative Drug Discovery, HKBU (Haimen) Institute of Science and Technology, Haimen 226100, China; 4 Shenzhen Lab of Combinatorial Compounds and Targeted Drug Delivery, HKBU Institute of Research and Continuing Education, Shenzhen 518000, China

**Keywords:** aptamers, purification, identification

## Abstract

Aptamers are single-stranded DNA or RNA oligonucleotides that can interact with target molecules through specific three-dimensional structures. The excellent features, such as high specificity and affinity for target proteins, small size, chemical stability, low immunogenicity, facile chemical synthesis, versatility in structural design and engineering, and accessible for site-specific modifications with functional moieties, make aptamers attractive molecules in the fields of clinical diagnostics and biopharmaceutical therapeutics. However, difficulties in purification and structural identification of aptamers remain a major impediment to their broad clinical application. In this mini-review, we present the recently attractive developments regarding the purification and identification of aptamers. We also discuss the advantages, limitations, and prospects for the major methods applied in purifying and identifying aptamers, which could facilitate the application of aptamers.

## 1. Introduction

The utilization of synthetic oligonucleotides is increasing in areas ranging from clinical diagnostics to novel biopharmaceutical therapeutics [1,2,3,4]. The automated synthesis of oligonucleotides is a highly efficient process with small amount of impurities produced at each step throughout the synthesis cycle [5,6]. Consequently, manufacturing organizations, as well as individuals who depend on the quality of the delivered oligonucleotides, have shown interest in developing effective and efficient ways to purify and analyze these important biological aptamers. For researchers, it is also necessary to work with oligonucleotides of higher purity. For this reason, oligonucleotides used for the purposes of gene knockout, genotying, and diagnosis typically need further purification after synthesis [7,8,9]. Failure to purify the crude synthetic oligonucleotides can seriously impede the ability of an organization or individual to achieve the desired results. Additionally, some modifications have been introduced, such as phosphorothioate linkages, incorporating modified nucleosides as 2′-OMe, 2′-OMOE, or 2′-F in the sequence, and attaching polyethylene glycol at the terminus to the structures of oligonucleotides in order to increase stability and bioavailability. However, these modifications also enhanced the challenges for analysis and purification [10,11,12].

A variety of techniques exist for the purification and isolation of aptamers from synthetic products to remove reaction products and unwanted oligonucleotides produced in the synthetic reactions [6]. The preparation of oligonucleotides is usually desalted, quantified, evaporated to dryness in a lyophilizer, and then supplied to the user in the form of power [13]. In this paper, we introduce the two main techniques, polyacrylamide gel electrophoresis (PAGE) and chromatography, which are widely used for the separation of target oligonucleotides [14,15].

The analysis of aptamers is the process to determine the conformation of the nucleic acid in a given oligonucleotide fragment. The structure analysis of the oligonucleotides had been applied in the fields of forensics and genotyping to elucidate structure features and functions.

Since the first structure (DNA double-helix) was elucidated in 1953 by Watson and Crick combining with the X-ray fiber diffraction data of Franklin and Wilkins, our knowledge about the advanced structure of DNA or RNA has developed immeasurably [16]. The morphologies of a whole range of nucleic acid double helical types were then subsequently established using fiber diffraction methods. More recently, due to the development of single-crystal and nuclear magnetic resonance (NMR) structural studies on defined-sequence oligonucleotides, more details about the advanced structure of nucleic acid have become increasingly understood [16,17,18]. However, the structures of nucleic acids continue to surprise researchers because of their existence in a wide variety of forms, such as left-handed and multiple-stranded helices [16,17]. Due to the development of X-ray crystallography, which could provide most of the highly-detailed structural information about nucleic acids, the determinations of nucleic acid structures has made many advances [19,20]. NMR spectroscopy, molecular modeling/simulation, and chemical/biochemical probe techniques also play important roles in providing information on structure, dynamics, and flexibility [17,18]. In this review, a brief introduction about the major methods studying oligonucleotides will be provided, emphasizing their scope, as well as their limitations for nucleic acid structural studies.

## 2. Separation and Purification of Oligonucleotides

### 2.1. Purification of Oligonucleotides by PAGE

PAGE separation can be conducted either at the analytical level or preparative scale and, in general, the principle of separating oligonucleotides by PAGE is based on their length, and thereby it offers enough resolution to differentiate full-length oligonucleotides from failed molecules [5]. Therefore, PAGE has been considered as a powerful tool for the separation of oligonucleotides. Polyacrylamide gels are formed by the polymerization of acrylamide in the presence of a cross-linking reagent, which is commonly *N*,*N*′-methylenebisacrylamide. The polymerization reaction produces a mesh-like network where long acrylamide fibers are cross-linked via bisacrylamide bridges. The size-sieving effect is the main factor that determines the separation properties of a polyacrylamide gel, wherein the relationship between the size of the pores and the size of the molecule determines the relative mobility of oligonucleotides through a polyacrylamide gel [7,8]. A variety of other important factors affect migration of oligonucleotides through the gels. It includes the conformation of the oligonucleotides, the voltage gradient, and the salt concentration of the buffer [5]. The pore size is mainly affected by two parameters: the concentration of acrylamide and the volume ratio of acrylamide and bisacrylamide [8]. The pore size decreases with increasing acrylamide concentration, thus it facilities the separation of smaller biomolecules [21]. The ratio of acrylamide to bisacrylamide affects the cross-linking frequency of the polyacrylamide mesh. For example, an increase in bisacrylamide concentration from 3.3% (acrylamide:bisacrylamide = 29:1 (*v*/*v*)) to 5% (acrylamide:bisacrylamide = 19:1 (*v*/*v*)) results in a decrease of the pore size, thus leading to a shift in the separation range toward smaller oligonucleotides [22]. A further increase in the concentration of bisacrylamide leads to an increase of the pore sizes because of non-uniform chain cross-linking. A 19:1 ratio of acrylamide to bisacrylamide is commonly used for the denaturing gel electrophoresis, while 29:1 is used for the native gel electrophoresis [5,21]. Denaturing polyacrylamide gels are also useful for preparative applications, such as small-scale purification of radioactive single-stranded probes and large-scale purification of synthetic oligonucleotides [23,24,25].

PAGE separation of long oligonucleotides more than 50–60 m are well-established and highly efficient [7], while the disadvantages are not negligible. The shortcomings of PAGE are mainly manifested in the following aspects: (1) Gels are typically overloaded for purification, which results in compromised resolution; (2) The 2′-OMe-, 2′-OMOE-, or 2′-F-modified nucleosides in the sequence cannot be separated by using PAGE because it resolves fragments by the differences in the length of single nucleotides; (3) The recovery rate of target oligonucleotides is low because the samples need to be further extracted from the gel and desalted; and (4) PAGE separation is a laborious and time-consuming process [6,7].

### 2.2. Purification of Oligonucleotides by Chromatographic Approaches

Chromatographic methods were used to analyze and purify the synthetic oligonucleotides since 1970s [26]. Significant developments in terms of instrument performance and stationary phase have been made during the past several years. Liquid chromatographic techniques, such as reverse-phase HPLC (RP-HPLC), ion-paired HPLC and ultra-performance liquid chromatography (UPLC) have been employed for analysis and purification of synthetic oligonucleotides [27].

#### 2.2.1. RP-HPLC

The principle of RP-HPLC is based on hydrophobicity between the packed material in the column and the compound, which means that the polar compounds are eluted first, while nonpolar compounds are retained in the column until more hydrophobic eluent is used [28]. When developing the methods to separate and purify oligonucleotides, some of the unique features of these molecules need to be considered. The pKa of the phosphodiester linkage is 2, which means that the oligonucleotides contain one negative charge for every phosphodiester linkage in an aqueous solution above pH 4. Therefore, traditional RP-LC conditions tend not to work well for the separation of oligonucleotides, and ion-exchange chromatography techniques or ion-paired RPLC need to be considered [29].

#### 2.2.2. Ion-Exchange Liquid Chromatography

Ion-exchange chromatography is an excellent method for separation of charged molecules, and it is commonly used to separate and purify multiple charged oligonucleotides [15,30]. With a salt-gradient elution mode on tertiary or quaternary ammonium-derivatized polymeric adsorbent or porous silica, the oligonucleotides could be separated by the ion-exchange chromatography method. Several kinds of advance structure, such as secondary, tertiary, dimers, or aggregates may exist in oligonucleotides, so denaturing conditions are required for these types of structures which could be achieved by adding organic solvents such ethanol or acetonitrile, using high-pH buffers or high temperature (55 to 65 °C depending on the column) [11,14,26].

However, the method of using higher-pH eluents cannot be applicable for analysis and purification of oligoribonucleotides/chimeric oligonucleotides with ribo-, 2′-OMe, and 2′-F nucleosides, because these structures are sensitive to higher pH conditions. Oligonucleotides of 2–30 m could be separated and purified successfully by anion-exchange chromatography based of the number of charged phosphate groups in the oligonucleotide backbone [10,14,26,27]. Purification of target oligonucleotides depends on their length, while yields of the oligonucleotides are usually sacrificed to obtaining better purity by heart-cutting the component of interest. Therefore, the collected fractions then are required to be desalted by RP-HPLC [15,30,31].

#### 2.2.3. Ion-Paired Reversed-Phase HPLC (IP-RP-HPLC)

IP-RP-HPLC is another very commonly used LC technique for the analysis and separation of oligonucleotides [32,33]. There are only slight differences between HPLC and IP-RP-HPLC. While the mechanism of HPLC separation relies solely on hydrophobicity, IP-RP-HPLC is an analytical technique in which a long-chained alkyl amine is added in low concentration to the mobile phase which interacts with negatively-charged oligonucleotides in order to achieve enhanced resolution [34,35,36]. Different mechanisms regarding on the ion pair interactions have been proposed which depend on whether the ion-pair process occurs in the mobile phase or not. Due to the presence of multiple charged species in the solvent mixture, the actual mechanism is certainly more complex. The retention and elution order are primarily governed by the factors, such as the charge of the oligonucleotides, the length of alkyl chain in the ion-pairing reagent and the proportion of organic solvent in the mobile phase [15,30]. Retention time could increase in proportion to the number of charges in the oligonucleotides. Increase of the hydrophobicity with the long alkyl chain in the ion-paring reagent leads to extended retention. Retention property in the column is reduced as the proportion of organic solvent increases [15,31,34,37]. The most powerful approach for optimizing separation resolution and selectivity is by changing the mobile phase [33,38]. Triethylammonium acetate (TEAA) is the most commonly used ion-paring buffer component [35,39], and a variety of other systems have been employed in IP-RP-HPLC for separation and purification of nucleosides and oligonucleotides, including tetrabutylammonium hydrogen sulfate, tetrabutylammonium phosphate, tetrabutylammonium bromide (TBAB), tributylammonium acetate (TBAA), tetrabutylammonium acetate, triethylammonium acetate (TEAB), hexylammonium acetate (HAA), and trimethylamine in combination with hexafluoroisopropanol (HFIP) [32,33,38,39,40]. Choosing HFIP and TEAB as ion-pairing agents is particularly attractive because it not only provides excellent separation, but is also compatible with MS coupling to HPLC [12,41]. In recent years, McCarthy et al. compared the usefulness of several ion-pairing systems in the separation of homo-oligonucleotide and hetero-oligonucleotide ladders [42]. McCarthy et al. [41] used TEAA, TEA/HFIP, TBAA, dimethylbutylammonium acetate (DMBAA), and HAA as ion-pairing reagents, respectively, in their experiment. With increasing concentration and hydrophobicity of the ion-pairing reagents the resolution of the homo-oligonucleotide ladder improved. With increasing oligonucleotides length and more hydrophobic ion-pairing reagents, such as HAA used in the purifying longer oligonucleotides (30–50 m), the peak capacity decreased. The separation is based predominantly on the charge-based mechanism in the separation of hetero-oligonucleotide ladders. Separations were improved by use of different ion-pairing systems ordered as: TEAA < DMBAA < TPAA < TEA/HFIP-HAA. Gilar et al. [27] utilized a UPLC system with RP columns packed with 1.7 µm sorbent to separate peptides, proteins, and oligonucleotides. They, respectively, measured retention factors, peak widths, and peak volumes of biopolymers under isocratic and gradient elution conditions. The results indicated that the flow rate of the Van Deemter optimum for 2.1 mm diameter columns packed with 1.7 µm porous sorbent was below 0.2 mL/min. The maximum peak capacity was achieved at flow rates between 0.15 and 1.0 mL/min, which depended on the molecular weight of the biopolymers. Column peak capacity was predicted as comprehensive functions of flow rate, gradient slope, and column length. Gilar et al. [15] developed a mathematical model for the prediction of oligonucleotide retention from sequence and length. They successfully selected the optimal initial gradient strength for fast HPLC purification of synthetic oligonucleotides, and also utilized an ion-paring mobile phase, comprised of TEA buffers by HFIP, which was useful for the highly efficient and less sequence-dependent separation of hetero-oligonucleotides. In 2013, Huang et al. [42] established a method using an XBridge IP-RP-HPLC column to separate and purify three RNA aptamers with 57, 58, and 59 nt by a single-nucleotide resolution, which conquered the limitation to RNA aptamers shorter than 25 nt. In 2014, they resolved and purified chemically-modified RNAs (2′-fluoro modified RNAs) with similar lengths using IP-RP-HPLC. Three 2′-fluoro-modified RNAs they used were F-U 57-nt, F-U 58-nt, and F-UC 58-nt. Their work also made it possible to separate RNA aptamers of similar sizes, but with different chemical modifications, such as uridine isomerization, nucleobase methylations, and hypermodified nucleotides in complex RNA maturation processes [10,43].

In contrast to anion-exchange liquid chromatography (AEC-HPLC) and ion exclusion chromatography (IEC-HPLC), IP-HPLC tends to be the most suitable method to separate and purify oligonucleotides in terms of buffers, sample concentration, and instrument requirements. Furthermore, another advantage of IP-HPLC is that it can be coupled to MS instrument directly, with which each individual peak could be identified easily, while IEC-HPLC or AEC-HPLC could not achieve that easily.

## 3. Methods for Studying Nucleic Acid Structure

### 3.1. Structural Determination by X-ray Crystallography Methods

To understand the functions and interactions of the biological macromolecules completely, to know their advanced structures is indispensable. In structural biology, X-ray crystallography is the most widely used technique and, without any size limitation, it could provide highly detailed structures of the biological macromolecules. X-rays have a wavelength of the same dimensions as interatomic bonds in molecules, which are about 1.5 Å. Diffraction or scattering of X-rays by molecules in ordered matter is the result of interactions between the electron distribution and the radiation of each atom [33,44]. Nucleic acid has two typical diffraction patterns, in the form of fibers and single crystals. By analysis of the scattered X-rays, analogous to a lens focusing scattered light from a microscope sample to reconstruct the internal molecular arrangement, could provide a picture of the electron density distribution in the molecule. During the diffraction process, the reconstruction is not generally straightforward because of the loss phase information from the individual reflected X-rays. The phase problem needs to be solved in order to calculate the electron density in three dimensions (as a Fourier series), which is commonly termed as a Fourier map [16,19,44,45]. The suitable equivalence of wavelength of X-ray and bond distance, is about 1–1.5 Å, which means that the electron density of individual atoms in a molecule could be resolved, and provided that the pattern of diffracted X-rays could be reconstituted into a real-space image [20,46].

The accuracy of determining the final structure of the nucleic acid results from a complex set of factors. First and foremost, to prepare and purify the chemically pure oligonucleotides molecules constitutes the major experimental bottleneck. Except for transfer RNAs or ribosomes, oligonucleotide molecules could not be obtained in sufficient quantities by extraction and purification from cultivated cells. Fortunately, based on molecular biology, chemical techniques and methods have progressed immensely in the last 15 years [47]. Secondly, to prepare the crystal diffracting at the highest possible resolution is a great experimental challenge which extremely demanding in patient manpower in order to derive reliable structural models [48]. Thirdly, the core of the crystallographic work is composed of data collection, treatment, the solution of the phasing problem, and refinement. In earlier times, the visual estimation of diffracted data was obtained using copper anodes which could present “zero-noise photon counting detectors” installed at novel generation synchrotron lines. In order to obtain accurate and cogent crystallographic structures that are biologically pertinent, the quality of the measured data and the appropriate choice of their mathematical treatment are both indispensable [49,50]. Finally, the derived structural models have to be discussed and assessed. In this part of the paper, we describe some methods which have been recently reported in the literature to overcome the four major difficulties in the progress of studying the structure of nucleic acids using X-ray crystallography [48,51].

Carrasco et al. [52] developed a method to synthesize nucleoside and oligonucleotide analogs containing selenium, which served as an anomalous scattering center to enable full name (MAD) phase determination in nucleotide X-ray crystallography. Du et al. [53] described the synthesis of oligonucleotides containing selenium at the 2′-α-position of uridine, and revealed crystallization and structural studies of the selenium-containing oligodeoxyribonucleotides. Mandal et al. [54] described racemic crystal structures of various DNA sequences and folded conformations, including duplexes, quadruplexes, and a four-way junction, showing that the advantages of racemic crystallography should be extended to DNA. Abdur et al. [46] reported a protein-nucleic acid complex structure determined by selenium-derivatized nucleic acids instead of the protein counterparts. They also found that the substrate-duplex conformation could play a significant role in guide-dependent RNA cleavage from the crystal structure. Furthermore, they observed that, in the presence of RNase H, the RNA scissile phosphate could form a hydrogen bond to the nucleophilic water molecule and position it in the structure for potential catalysis. The unique Se atom-specific mutagenesis (SAM) of nucleic acids in their experiment had opened a new avenue for not only structure determination, but also mechanistic studies of protein-nucleic acid complex. Vasilyev and Serganov [55] employed the in vitro transcription methodology to prepare oligoribonucleotide less than 5 m for crystallographic and biochemical studies. Zhang et al. [56] have successfully developed a synthetic strategy of selenium-derivatized nucleic acids (SeNA) for nucleic acid crystallography in order to overcome the major bottleneck of crystallization and phase determination in crystallography. Peselis et al. [57] outlined the methodology to synthesize riboswitch RNAs and prepared riboswitch-ligand complexes for crystallographic and biochemical studies. They also described how to design, prepare, and conduct crystallization screening of riboswitch-ligand complexes. Dégut et al. [58] described two methods to produce and purify tRNA by performing X-ray studies. Ferré-D’Amaré [59] provided detailed methods for the in vitro transcription of an engineered tetracycline aptamer RNA and its co-crystallization with U1A as an example of RNA structure determination, which was facilitated by UIA, both for the preparation of well-ordered crystals and for experimental phase determination. Sherman et al. [60] described Fab chaperone-assisted RNA crystallography (CARC), a systematic technique to increase the success of RNA crystallography by facilitating crystal packing, as well as expediting phase determination through themolecular replacement of conserved Fab domains. Egli and Pallan [61] described an alternative approach to determine the crystal structures of the dodecamer in case molecular replacement does not produce a solution, or crystals of DNA alone cannot be grown. This method was based on the discovery that many DNA dodecamers could be readily co-crystallized with Bacillus halodurans RNase H, whereby the enzyme was unable to cleave the DNA. Diederichs [62] discussed the relationship between precision and accuracy and the crystallographic indicators, as well as topics like completeness and high-resolution cutoff. These concepts were applied in the context of presenting good practices for data processing with a widely used package, XDS. They also gave recommendations for how to minimize the impact of several typical problems, like ice rings and shaded areas. Finke et al. [63] optimized the strategies for collecting data with high-quality for experimental phasing, particularly emphasizing on minimizing errors from radiation damage, as well as the instrument in detail. They also emphasized data processing for “on-the-fly” decision-making during data collection, a critical process when data quality depends directly on information gathered while at the synchrotron. Zubieta and Nanao [64] provided a practical set of data collection and processing strategies for phasing macromolecular structures using radiation damage-induced phasing or “RIP”. Batey and Kieft [65] developed a reliable means to use hexamine cations to address the challenge that many of tools available for solving protein structures but which are not available for RNA. The process involved with engineering the RNA to introduce a reliable hexamine binding site into the structure, then soaking crystals of these RNAs with iridium (III) or cobalt (III) compounds in a “directed soaking” strategy. Diffraction data obtained from these crystals then could be used in SAD (Single-wavelength anomalous diffraction) or MAD phasing.

### 3.2. Structural Determination by NMR

NMR is an incredibly powerful technique because of its broad dynamic range and the quantitative and nondestructive features. Furthermore, the versatility that accompanies NMR’s ability to provide atomic resolution is readily apparent for the study of oligonucleotides. NMR methods enable structures to be determined in solution, largely by means of measuring the proton–proton coupling constants and through-space nuclear Overhauser effect (NOE) derived distance using 2D NMR methods [17,18]. The obvious advantage of solution-phase studies is that molecules do not have to be crystalized, which is the major limitation for X-ray crystallography to analyze oligonucleotides. In this part of the review, we will introduce the NMR method applied to the structure determination of nucleic acid, and strive to describe how NMR is being used to analyze the structure of oligonucleotides. This paper will also summarize the characteristics and limitations of NMR for analysis of the structures of oligonucleotides.

#### 3.2.1. Overview

In 1983, Reid et al. used a two-dimensional spectrum to identify an oligonucleotide containing 10 base-pairs for the first time [66]. In 1986, Wuthrich discussed the method of studying the structure of DNA by NMR in his famous book “NMR of Proteins and Nucleic Acids” in detail, and now this book is still the guide for the researchers who study the structure of DNA [67]. In 1998, Dingley and Grzesiek firstly directly observed the hydrogen bonds in RNA base pairs by internucleotide ^2^J NN couplings [68]. This research was quickly applied to DNA. Pervushin [69] described the NMR observation of ^15^N–^15^N and ^1^H–^15^N scalar couplings across the hydrogen bonds in Watson–Crick base pairs in a DNA duplex, ^h^J NN and ^h^J NH using two-dimensional [^15^N,^1^H]-transverse relaxation-optimized spectroscopy (TROSY) with a ^15^N-labeled 14 nt DNA duplex to measure ^h^J NN and the two-dimensional ^h^J NN-correlation-[^15^N,^1^H]-TROSY experiment to correlate the chemical shifts of pairs of hydrogen bond-related ^15^N spins and to observe, for the first time, ^h^J NH scalar couplings with values in the range 2–3.6 Hz. As we all know, because of the hydrogen bonds in nucleic acid base pairs, the two-dimensional structure of the nucleic acid could be stable. The hydrogen bonds existing in DNA have drawn great interest in studying the structures of nucleic acid [69,70].

Recently, a large number of experiments have been established to provide much information on the structures of protein–DNA, protein–RNA, and drug–DNA complexes [71,72,73]. Anderson et al. [74] developed a chemical approach for site-specific identification of NMR signals from protein side-chain NH^3+^ groups forming intermolecular ion pairs in protein-nucleic acid complexes. Wolter et al. currently determined the structure of a GTP-aptamer—the so-called class II aptamer—bound to GTP using NMR spectroscopy in solution. As a prerequisite for a full-structure determination, ^15^N, ^1^H, ^13^C, and practical ^31^P-NMR resonance assignments for the class II GTP-aptamer bound to GTP has been reported [75].

#### 3.2.2. Chemical Shift Distribution of Oligonucleotide Protons

**Figure 1:**
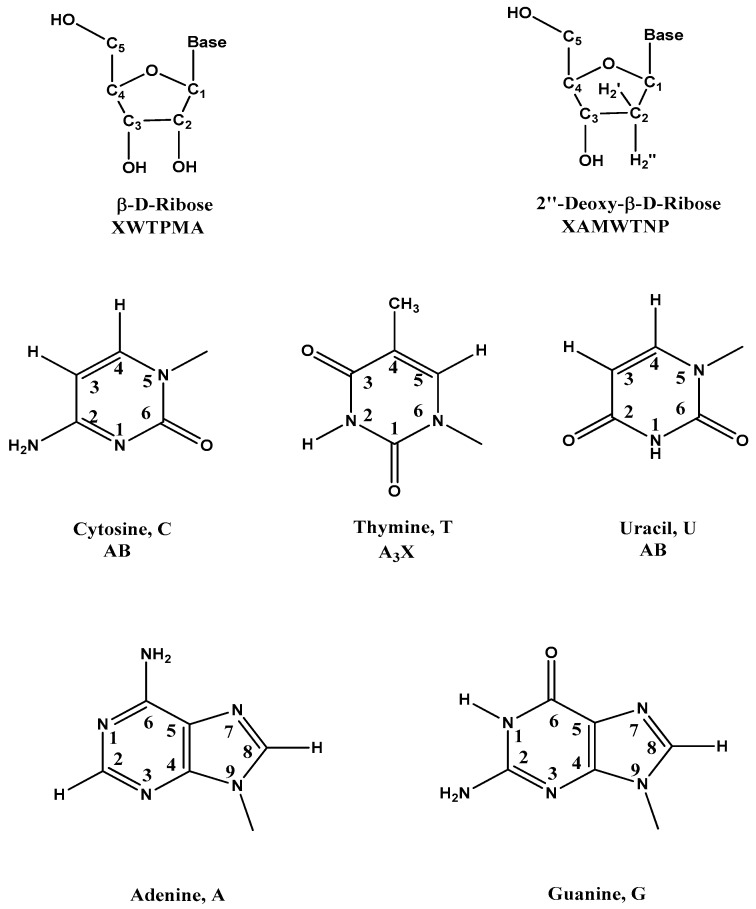
The structures, symbols, and ^1^H spin systems of the β-d-riboses, 2′′-deoxy-β-d-riboses, and five common bases in DNA and RNA. (Modified according to the reference [67]).

The basic of NMR is that some nuclei could be oriented by a strong magnetic field and then absorb radiation that meets the frequencies characteristic of that nuclei. The oriented nuclei are irradiated through a radio frequency field (FR) which is a specific duration and strength to rotate the magnetization from the *z* axis into the transverse (*xy*) plane [18,67]. The frequency of the radiation necessary for absorption of energy depends on both the chemical environment and type of nucleus. In the transverse plane, the magnetization decays with time, and with the individual nuclei precessing at different rates, which depends on their electronic and chemical environment. An electrical current is detected through the precession, which also produces the free induction decay (FID) [6]. Using Fourier transformation of the FID, the NMR spectrum is obtained. In different environments, each nuclei of the same element cause distinct spectral lines (also called chemical shift), which makes it possible to observe signals from individual atoms [6]. The ability to achieve atomic resolution makes NMR such a powerful technique. Figure 1 shows the structures, symbols, and ^1^H spin systems of the β-d-riboses and the five common bases in DNA and RNA. The approximate ranges of proton chemical shifts for oligonucleotides are shown in Table 1 [17,18,67].

#### 3.2.3. NMR Experiment Considerations

##### Sample Preparation

Many of the therapeutically-based oligonucleotides need to be highly amenable to NMR study. Common NMR sample preparation conditions are 1 mM oligonucleotide in 10–20 mM phosphate buffer (pH range of 4.5 to 7) and 50–200 mM NaCl. NMR oligonucleotide sample conditions in research also commonly include a small amount of MgCl_2_ and/or EDTA (Ethylenediaminetetraacetic acid disodium salt). Other buffers can be used, such as preferably-deuterated buffers for proton detection [76]. Souard et al. developed an approach based on the design-of-experiments methodology in order to overcome the problem that short oligonucleotides are intrinsically flexible, which renders their active conformation highly sensitive to the NMR experimental conditions [77]. Higher buffer concentrations, which may catalyze the hydrogen exchange of imino protons, therefore, should be kept low. The sensitivity and resolution have been decreased because of the exchange of imino protons resulting in broadened resonances [76]. The data collection of oligonucleotides is typically performed when the sample is in 100% D_2_O, when it is necessary to do assignments other than the imino resonances. The exchangeable proton would not be observed in the ^1^H NMR spectrum since it has been exchanged with deuterium. Due to this, the complexity of the spectra has been decreased, and the differentiation between exchangeable and nonexchangeable protons would be confirmed. In addition, the spectra could have better resolution due to the slightly different dynamic properties of deuterium oxide relative to H_2_O [76].

##### Gradient Suppression Technique

It is often necessary to obtain proton spectra from samples dissolved in non-deuterated solvents in biological NMR. In order to observe the imino protons in oligonucleotides, the experiment is typically conduced in H_2_O with a small amount of deuterium oxide for a lock [76]. In this experiment, the exchangeable protons, such as imino and amino protons in RNA/DNA could be detected. The high proton concentration in the non-deuterated solvent would generate an NMR signal over a thousand times more than any of the solute signals. To overcome this problem, solvent suppression techniques should be used during data acquisition [67,78]. The simplest method is to pre-saturate the residual water, but the relative fast exchange of imino/amino resonances with water leads to saturation of their resonances by the transfer of saturation. Therefore, if we are not interested in the imino/amino resonances, presaturation could be used. Many selective excitation and solvent suppression techniques exist, such as jump return and Watergate, to deduce the large signal from the water. Utilizing WET sequence makes it possible to suppress multiple signals [79].

##### NMR Experiments

For studying the structure of therapeutic-based oligonucleotides using NMR, methods must rely on naturally abundant nuclei, such as protons, carbon, phosphorous, and fluorine [17,67]. A summary of common NMR experiments used in studying the structure of oligonucleotides is displayed in Table 2.

To observe the scalar or the bond connectivity between neighboring protons, the methods COSY (correlation spectroscopy) and TOCSY (Total Correlation spectroscopy) are used [80]. Known as the spin system, the TOCSY-type spectrum contains cross peaks for all protons connected by carbon atoms, whereas the COSY-type spectrum contains cross peaks between protons separated by three bonds. Correlating proton lines with carbons connected by one bond, the ^13^C-HSQC NMR experiment makes it possible to assign the protonated carbons directly [78,80]. In addition, other scalar couplings, such as long-range ^1^H-^31^P correlations in the two-dimensional heteronuclear correlation spectroscopy (HETCOR), could be used between NMR active nuclei [81]. In order to aid in the assignment of resonances, the scalar coupling could be utilized, but as the magnitude of coupling between vicinal resonances is dependent on the dihedral angles between the two atoms, they also could yield quantitative information about bond angles.

To measure internuclear distances, the dipolar interaction of two NMR active nuclei could be utilized, which is the cause of the nuclear Overhauser effect (NOE) [76]. In the two-dimensional NOESY NMR spectrum, through-space connections between protons in close proximity to each other could be observed. It is possible to observe the correlation for protons within approximately a 5 Å range, on the basis of the choice of instrument parameters. Assigning the chemical shifts, the information is invaluable. To measure the NOESY correlations makes it possible to obtain distance restraints for NMR structure determination, with care in the choice of NMR acquisition parameters.

Applications of NMR to study the structure and dynamics of the oligonucleotides are reviewed in aspects regarding NMR peak assignments, NMR constraints, structure calculation, main features of oligonucleotide NMR spectra, and dynamics in solution [72,82,84]. However, there are also many unsolved problems in oligonucleotide NMR studies, including the following aspects: (1) In the molecular modeling force field, the dihedral angle parameters are not usually consistent with those in irregular aptamers [101]; (2) The reproducibility is usually not good for aptamers structures simulated by molecular modeling [102]; (3) The ring current theory used for explaining aptamer chemical shifts is yet not ready for use [103]; (4) The model-free model applied for interpreting steady-state NOE still has some difficulties; and (5) Due to resonance overlap, the analysis of the structure of more than 30-m aptamers can be rather difficult using the NMR method. Nonetheless, the structural and dynamical information we obtain from NMR technology still plays an important role in analyzing the structure of biomacromolecules, such as aptamers, proteins, and aptamer–protein complex. As the therapeutic utility of oligonucleotides develops, it is accordingly expected that new and novel NMR methodologies will be applied to meet the increasing challenges.

## 4. Conclusions

The purification of oligonucleotides using HPLC could save time and make the process of purification relatively easy and straightforward, which has been the standard for quick and efficient large-scale purification and separation from crude synthetic mixtures.

To understand the biological function of the aptamer, we must know its structural features. Since oligonucleotides interact with proteins in all of their metabolic or control operations, specific and mutual recognition of the two or more reactants is required. It is presupposed that the partners involved have well-defined three-dimensional structures which, if we desire to understand the function at the atomic level, we must also know the structure of oligonucleotides at the atomic level. Furthermore, with the tertiary molecular structures of some aptamer/protein-target complexes being elucidated, the aptamer–protein interaction has been analyzed at the molecular levels, which indicates that non-covalent interactions formed between specific nucleic acids and target conformations, including stacking interactions, van der Waals contacts, and hydrogen bonding. The drug design and drug screening would be facilitated more quickly with the elucidation of aptamer and aptamer–protein interaction.

## Figures and Tables

**Table 1:** Approximate ^1^H chemical shift ranges in single-stranded and duplex DNA and RNA fragments. (Modified according to the reference [67]).

Code	δ (ppm)	Comments
**β-d-Riboses**		
2′	1.8–3.0	2′, 2′′ H in DNA
4′	3.7–4.5	4′, 5′, 5′′ H in DNA
3′	4.4–5.2	3′ H in DNA
2′, 3′, 4′, 5′, 5′′	3.7–5.2	2′, 3′, 4′, 5′, 5′′ H in RNA
1′	5.3–6.3	1′ H in DNA and RNA
**Five Common Bases**		
Me	1.2–1.6	Me of T
5	5.3–6.0	5 H of C and U
6	7.1–7.6	6 H of C, T, and U
2, 8	7.3–8.4	8 H of A and G, 2 H of A
–NH_2_	6.6–9.0	NH_2_ of A, U And G
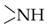	10–15	NH of G, T and U

**Table 2:** NMR experiments used in studying the structure of oligonucleotides (Modified and updated according to the reference [76]).

Stage	NMR Experiments	Information	References
Identification (fingerprint)	^1^H ^31^P or ^19^F 1D; ^1^H 1D temperature studies	Determine the presence of secondary structure, including the number of base pairs; Determine the secondary structure	[68,69,82,83,84]
Impurity or conformational identification	^1^H ^31^P ^19^F 1D; LC-NMR or DOSY	Identify and/or quantitate the presence of multiple conformations and/or impurities	[85,86,87]
Imino assignment	2D NOESY; 2D TOCSY/COSY	Identify base pairs and show connectivity (imino walk)	[67,68,69,75,88]
Base proton assignment of aromatic walk	^13^C-HSQC; D_2_O NOESY; D_2_O TOCSY	Secondary structure; Aromatic to H1′ sugar sequential assignment (walk); H6 T/C and H8 G/A base assignments	[86,89,90,91]
Complete ^1^H assignment (as possible); ^13^C assignment (as possible)	Different temperatures and/or mixing times may be necessary (NOESY and TOCSY)	sugar assignment as possible; Additional base protons (e.g., H2 of A bases); Carbon assignments as possible	[78,92,93]
Tertiary structure (rough)	Quantitative NOESY; 2D ^1^H ^31^P HETCOR; Long-range heteronuclear correlation experiments (HMBC, HSQMBC, J-quantitative J-resolved experiments)	Distance restraints from full assignment of the correlations observed in the two-dimensional NOESY spectra; Angle restraints from quantitative analysis of J coupling constant	[73,90,91,94]
Tertiary structure (refined)	1D and/or 2D NMR spectra in aligning media	residual dipolar couplings utilized to obtain long-range restraints	[83,95]
Dynamics	1D or 2D T1 or T2 experiments such as inversion recovery and CPMG	Sequence dependence dynamics	[88,93,96,97,98]
Ligand interaction	Observation of chemical shift perturbation in NMR experiments or cross-correlation experiments; Isotope-filtered NMR methods; STD NMR methods; F-Site-specific-Labeled Nucleotides	Oligonucleotide resonances in contact with ligand (protein)	[71,74,75,84,86,99,100]

## Abbreviations

PAGE	polyacrylamide gel electrophoresis
NMR	nuclear magnetic resonance
RP-HPLC	reverse-phase HPLC
UPLC	ultra-performance liquid chromatography
IP-RP-HPLC	Ion-Paired Reversed-Phase HPLC
TEAA	Triethylammonium acetate
TBAB	tetrabutylammonium bromide
TBAA	tributylammonium acetate
TEAB	triethylammonium acetate
HAA	hexylammonium acetate
HFIP	hexafluoroisopropanol
DMBAA	dimethylbutylammonium acetate
AEC-HPLC	anion-exchange liquid chromatography
IEC-HPLC	ion exclusion chromatography
SeNA	selenium-derivatized nucleic acids
CARC	chaperone assisted RNA crystallography
NOE	Overhauser effect
TROSY	transverse relaxation-optimized spectroscopy
FID	free induction decay
COSY	Correlation Spectroscopy
TOCSY	Total Correlation Spectroscopy
HSQC	Heteronuclear Single Quantum Coherence
HETCOR	Heteronuclear Correlation Spectroscopy
SAM	Se atom-specific mutagenesis
CPMG	Carr-Purcell-Meiboom-Gill
AEC-HPLC	anion-exchange liquid chromatography
IEC-HPLC	ion exclusion chromatography
SAD	Single-wavelength anomalous diffraction

## References

[1] Sun H., Zhu X., Lu P.Y., Zu L.Y. (2014). Oligonucleotide aptamers: New tools for targeted cancer therapy. Mol. Ther. Nucleic Acids.

[2] Zhao N., You J., Zeng Z., Zu L.Y. (2013). An ultra pH-sensitive and aptamer-equipped nanoscale drug—Delivery system for selective killing of tumor cells. Small.

[3] Lee J.W., Kim H.J., Heo K. (2015). Therapeutic aptamers: Developmental potential as anticancer drugs. BMB Rep..

[4] Zhu G., Ye M., Donovan M.J. (2012). Nucleic acid aptamers: An emerging frontier in cancer therapy. Chem. Commun. (Camb.).

[5] Lopez-Gomollon S., Nicolas F.E. (2013). Purification of DNA oligos by denaturing polyacrylamide gel electrophoresis (PAGE). Methods Enzymol..

[6] Cramer H., Finn K.J., Herzberg E. (2011). Purity analysis and impurities determination by reversed-phase high-performance liquid chromatography. Handbook of Analysis of Oligonucleotides and Related Products.

[7] Petrov A., Tsa A., Puglisi J.D. (2013). Analysis of RNA by analytical polyacrylamide gel electrophoresis. Methods Enzymol..

[8] Petrov A., Wu T., Puglisi E.V. (2013). RNA purification by preparative polyacrylamide gel electrophoresis. Methods Enzymol..

[9] Holmes D.L., Stellwagen N.C. (1991). Estimation of polyacrylamide gel pore size from Ferguson plots of linear DNA fragments. II. Comparison of gels with different crosslinker concentrations, added agarose and added linear polyacrylamide. Electrophoresis.

[10] Ali W.H., Pichon V. (2014). Characterization of oligosorbents and application to the purification of ochratoxin A from wheat extracts. Anal. Bioanal. Chem..

[11] Abeydeera N.D., Egli M., Cox N., Yang Y.B. (2016). Evoking picomolar binding in RNA by a single phosphorodithioate linkage. Nucleic Acids Res..

[12] Gilar M. (2001). Analysis and purification of synthetic oligonucleotides by reversed-phase high-performance liquid chromatography with photodiode array and mass spectrometry detection. Anal. Biochem..

[13] Zlobina M. (2016). Efficient large-scale preparation and purification of short single-stranded RNA oligonucleotides. Biotechniques.

[14] Gilar M., Bouvier E.S.P. (2000). Purification of crude DNA oligonucleotides by solid-phase extraction and reversed-phase high-performance liquid chromatography. J. Chromatogr. A.

[15] Gilar M., Fountain K.J., Budman Y. (2002). Ion-pair reversed-phase high-performance liquid chromatography analysis of oligonucleotides. Retention prediction. J. Chromatogr. A.

[16] Westhof E., Ennifar E. (2016). Perspectives and pitfalls in nucleic acids crystallography. Nucleic Acid Crystallography: Methods and Protocols.

[17] Arnott S., Neidle S. (1999). Polynucleotide secondary structures: An historical perspective. Oxford Handbook of Nucleic Acid Structure.

[18] Neidle S. (2008). DNA structure as observed in fibers and crystals. Principles of Nucleic Acid Structure.

[19] Messerschmidt A. (2007). X-ray Crystallography of Biomacromolecules: A Practical Guide.

[20] Blackburn G.M. (2006). Nucleic Acids in Chemistry and Biology.

[21] Holmes D.L., Stellwagen N.C. (1991). Estimation of polyacrylamide gel pore size from Ferguson plots of normal and anomalously migrating DNA fragments I. Gels containing 3% *N*,*N*′-methylenebisacrylamide. Electrophoresis.

[22] Greenough L. (2016). Adapting capillary gel electrophoresis as a sensitive, high-throughput method to accelerate characterization of nucleic acid metabolic enzymes. Nucleic Acids Res..

[23] Stellwagen N.C. (2009). Electrophoresis of DNA in agarose gels, polyacrylamide gels and in free solution. Electrophoresis.

[24] Greene J.M., Struhl K. (2001). S1 analysis of messenger RNA using single-stranded DNA probes. Curr. Protoc. Mol. Biol..

[25] Gilman M. (1993). Ribonuclease protection assay. Curr. Protoc. Mol. Biol..

[26] Andrus A., Bloch W. (1998). HPLC of oligonucleotides and polynucleotides. HPLC Macromol..

[27] Gilar M., Neue U.D. (2007). Peak capacity in gradient reversed-phase liquid chromatography of biopolymers. Theoretical and practical implications for the separation of oligonucleotides. J. Chromatogr. A.

[28] Lin C.Y., Huang Z., Jaremko W. (2014). High-performance liquid chromatography purification of chemically modified RNA aptamers. Anal. Biochem..

[29] Biba M., Mao B., Welch C.J. (2014). Liquid chromatography methods for the separation of short RNA oligonucleotides. LCGC N. Am..

[30] Fountain K.J., Gilar M., Budman Y. (2003). Purification of dye-labeled oligonucleotides by ion-pair reversed-phase high-performance liquid chromatography. J. Chromatogr. B.

[31] Dickman M.J. (2011). Ion pair reverse-phase chromatography: A versatile platform for the analysis of RNA. Chromatogr. Today.

[32] Hoffman N.E., Liao J.C. (1977). Reversed phase high performance liquid chromatographic separations of nucleotides in the presence of solvophobic ions. Anal. Chem..

[33] Andrus A., Kuimelis R.G. (2015). Analysis and purification of synthetic nucleic acids using HPLC. Curr. Protoc. Nucleic Acid Chem..

[34] Bidlingmeyer B.A., Deming S.N., Price W.P. (1979). Retention mechamism for reversed-phase ion-pair liquid chromatography. J. Chromatogr. A.

[35] Melander W.R., Horváth C. (1980). Mechanistic study on ion-pair reversed-phase chromatography. J. Chromatogr. A.

[36] Cecchi T. (2008). Ion pairing chromatography. Crit. Rev. Anal. Chem..

[37] Agrawal S., Tang J.Y., Brown D.M. (1990). Analytical study of phosphorothioate analogues of oligodeoxynucleotides using high-performance liquid chromatography. J. Chromatogr. A.

[38] Haupt W., Pingoud A. (1983). Comparison of several high-performance liquid chromatography techniques for the separation of oligodeoxynucleotides according to their chain lengths. J. Chromatogr. A.

[39] McKeown A.P., Shaw P.N., Barrett D.A. (2002). Retention behaviour of an homologous series of oligodeoxythymidilic acids using reversed-phase ion-pair chromatography. Chromatographia.

[40] Ikuta S., Chattopadhyaya R., Dickerson R.E. (1984). Reverse-phase polystyrene column for purification and analysis of DNA oligomers. Anal. Chem..

[41] McCarthy S.M., Warren W.J., Dubey A., Gilar M. Ion-pairing systems for reversed-phase chromatography separation of oligonucleotides. Presented at TIDES Conference.

[42] Huang Z., Jayaseelan S., Hebert J. (2013). Single-nucleotide resolution of RNAs up to 59 nucleotides by high-performance liquid chromatography. Anal. Biochem..

[43] McCarthy S.M., Gilar M., Gebler J. (2009). Reversed-phase ion-pair liquid chromatography analysis and purification of small interfering RNA. Anal. Biochem..

[44] Arrhenius G. (1955). X-ray diffraction procedures for polycrystalline and amorphous materials. J. Chem. Educ..

[45] Dauter Z., Adamiak D.A. (2001). Anomalous signal of phosphorus used for phasing DNA oligomer: Importance of data redundancy. Acta Crystallogr. D Biol. Crystallogr..

[46] Abdur R., Gerlits O.O., Gan J. (2014). Novel complex MAD phasing and RNase H structural insights using selenium oligonucleotides. Acta Crystallogr. D Biol. Crystallogr..

[47] Buschmann D., Haberberger A., Kirchner B. (2016). Toward reliable biomarker signatures in the age of liquid biopsies—How to standardize the small RNA-Seq workflow. Nucleic Acids Res..

[48] Mandal P.K., Chandrasekaran A.R., Madhanagopal B.R. (2012). Ring crystals of oligonucleotides: Growth stages and X-ray diffraction studies. J. Cryst. Growth.

[49] Narayanan B.C., Westbrook J., Ghosh S. (2014). The nucleic acid database: New features and capabilities. Nucleic Acids Res..

[50] Daubner G.M., Cléry A., Allain F.H.T. (2013). RRM-RNA recognition: NMR or crystallography and new findings. Curr. Opin. Struct. Biol..

[51] Lipfert J. (2014). Understanding nucleic acid-ion interactions. Annu. Rev. Biochem..

[52] Carrasco N. (2001). Synthesis of selenium-derivatized nucleosides and oligonucleotides for X-ray crystallography. Nucleos Nucleot Nucl..

[53] Du Q., Carrasco N., Teplova M. (2002). Internal derivatization of oligonucleotides with selenium for X-ray crystallography using MAD. J. Am. Chem. Soc..

[54] Mandal P.K., Collie G.W., Kauffmann B. (2014). Racemic DNA crystallography. Angew. Chem..

[55] Vasilyev N., Serganov A. (2016). Preparation of short 5′-triphosphorylated oligoribonucleotides for crystallographic and biochemical studies. Nucleic Acid Crystallogr. Methods Protoc..

[56] Zhang W., Szostak J.W., Huang Z. (2016). Nucleic acid crystallization and X-ray crystallography facilitated by single selenium atom. Front. Chem. Sci. Eng..

[57] Peselis A., Gao A., Serganov A. (2015). Preparation and crystallization of riboswitches. Nucleic Acid Crystallogr. Methods Protoc..

[58] Dégut C. (2015). In vitro/in vivo production of tRNA for X-ray studies. Methods Mol. Biol..

[59] Ferré-D’Amaré A.R. (2016). Use of the U1A Protein to facilitate crystallization and structure determination of large RNAs. Methods Mol. Biol..

[60] Sherman E., Archer J., Ye J.D. (2016). Fab chaperone—Assisted RNA crystallography (Fab CARC). Methods Mol. Biol..

[61] Egli M., Pallan P.S. (2016). Generating crystallographic models of DNA dodecamers from structures of RNase H:DNA complexes. Nucleic Acid Crystallogr. Methods Protoc..

[62] Diederichs K. (2016). Crystallographic data and model quality. Nucleic Acid Crystallogr. Methods Protoc..

[63] Finke A.D., Panepucci E., Vonrhein C. (2016). Advanced crystallographic data collection protocols for experimental phasing. Nucleic Acid Crystallogr. Methods Protoc..

[64] Zubieta C., Nanao M.H. (2016). Practical radiation damage-induced phasing. Nucleic Acid Crystallogr. Methods Protoc..

[65] Batey R.T., Kieft J.S. (2016). Soaking hexammine cations into RNA crystals to obtain derivatives for phasing diffraction data. Nucleic Acid Crystallogr. Methods Protoc..

[66] Hare D.R., Wemmer D.E., Chou S.H. (1983). Assignment of the non-exchangeable proton resonances of d(CGCGAATTCGCG) using two-dimensional nuclear magnetic resonance methods. J. Mol. Biol..

[67] Wuthrich K. (1986). NMR of Proteins and Nucleic Acids.

[68] Dingley A.J., Grzesiek S. (1998). Direct observation of hydrogen bonds in nucleic acid base pairs byInternucleotide 2 J NN couplings. J. Am. Chem. Soc..

[69] Pervushin K., Ono A., Fernández C. (1998). NMR scalar couplings across Watson–Crick base pair hydrogen bonds in DNA observed by transverse relaxation-optimized spectroscopy. Proc. Natl. Acad. Sci. USA.

[70] Liu A., Majumdar A., Hu W. (2000). NMR detection of NHOC hydrogen bonds in ^13^C,^15^N-labeled nucleic acids. J. Am. Chem. Soc..

[71] Zwahlen C., Legault P., Vincent S.J.F. (1997). Methods for measurement of intermolecular NOEs by multinuclear NMR spectroscopy: Application to a bacteriophage λ N-Peptide/boxB RNA complex. J. Am. Chem. Soc..

[72] Lam S.L., Ip L.N. (2002). Low temperature solution structures and base pair stacking of double helical d(CGTACG) 2. J. Biomol. Struct. Dyn..

[73] Parella T., Espinosa J.F. (2013). Long-range proton-carbon coupling constants: NMR methods and applications. Prog. Nucl. Magn. Reson. Spectrosc..

[74] Anderson K.M., Nguyen D., Esadze A. (2015). A chemical approach for site-specific identification of NMR signals from protein side-chain NH^3+^ groups forming intermolecular ion pairs in protein–nucleic acid complexes. J. Biomol. NMR.

[75] Wolter A.C., Duchardt-Ferner E., Nasiri A.H. (2016). NMR resonance assignments for the class II GTP binding RNA aptamer in complex with GTP. Biomol. NMR Assign..

[76] DeRider M.L., Brooks D., Burt G. (2011). Structural Determination by NMR. Handbook of Analysis of Oligonucleotides and Related Products.

[77] Souard F., Perrier S., Noël V. (2015). Optimization of experimental parameters to explore small-ligand/aptamer interactions through use of ^1^H NMR spectroscopy and molecular modeling. Chemistry.

[78] Latham M.P., Brown D.J., McCallum S.A. (2005). NMR methods for studying the structure and dynamics of RNA. Chembiochem.

[79] Zheng G., Price W.S. (2010). Solvent signal suppression in NMR. Prog. Nucl. Magn. Reson. Spectrosc..

[80] Markley J.L., Bax A., Arata Y. (1998). Recommendations for the presentation of NMR structures of proteins and nucleic acids. J. Mol. Biol..

[81] Thibaudeau C., Chattopadhyaya J. (2006). The discovery of intramolecular stereoelectronic forces that drive the sugar conformation in nucleosides and nucleotides. Nucleos Nucleot Nucl..

[82] Gossert A., Jahnke W. (2016). NMR in drug discovery: A practical guide to identification and validation of ligands interacting with biological macromolecules. Prog. Nucl. Magn. Reson. Spectrosc..

[83] Mukhopadhyay R. (2007). Liquid NMR probes: Oh so many choices. Anal. Chem..

[84] Imeddourene A.B., Xu X., Zargarian L. (2016). The intrinsic mechanics of B-DNA in solution characterized by NMR. Nucleic Acids Res..

[85] Feigon J., Skelenář V., Wang E. (1992). ^1^H NMR spectroscopy of DNA. Methods Enzymol..

[86] Scott L.G., Hennig M. (2016). F-site-specific-labeled nucleotides for nucleic acid structural analysis by NMR. Methods Enzymol..

[87] Graber D., Moroder H., Micura R. (2008). ^19^F NMR spectroscopy for the analysis of RNA secondary structure populations. J. Am. Chem. Soc..

[88] Shajani Z., Varani G. (2007). NMR studies of dynamics in RNA and DNA by ^13^C relaxation. Biopolymers.

[89] Schmitz U., Ulyanov N.B., Kumar A. (1993). Molecular dynamics with weighted time-averaged restraints for a DNA octamer, dynamic interpretation of nuclear magnetic resonance data. J. Mol. Biol..

[90] Borden K.L.B., Jenkins T.C., Skelly J.V. (1992). Conformational properties of the G.G mismatch in d(CGC-GAATTGGCG) 2 determined by NMR. Biochemistry.

[91] Ulyanov N.B., Bauer W.R., James T.L. (2002). High resolution NMR structure of an AT-rich DNA sequence. J. Biomol. NMR.

[92] Lipari G., Szabo A. (1982). Model-free approach to the interpretation of nuclear magnetic resonance relaxation in macro-molecules. 1. Theory and range of validity. J. Am. Chem. Soc..

[93] Kojima C., Ono A., Kainosho M. (1998). DNA duplex dynamics: NMR relaxation studies of a decamer with uniformly ^13^C labeled purine nucleotides. J. Magn. Reson..

[94] Borgias B.A., James T.L. (1990). Procedure for matrix analysis of relaxation for discerning geometry of an aqueous structure. J. Magn. Reson..

[95] Wang E., Malek S., Feigon J. (1992). Structure of a G.T.A triplet in an intramolecular DNA triplex. Biochemistry.

[96] Bax A., Kontaxis G., Tjandra N. (2001). Dipolar couplings in macromolecular structure determination. Methods Enzymol..

[97] Spielmann H.P. (1998). Dynamics in psoralen-damaged DNA by ^1^H detected natural abundance ^13^C NMR spectroscopy. Biochemistry.

[98] Eimer W., Williamson J.R., Boxer S.G. (1990). Characterization of the overall and internal dynamics of short oligonu-cleotides by depolarized dynamic light-scattering and NMR relaxation measurements. Biochemistry.

[99] Breeze A.L. (2000). Isotope-filtered NMR methods for the study of biomolecular structure and interactions. Prog. Nucl. Magn. Reson. Spectrosc..

[100] Wang Y.S., Liu D., Wyss D.F. (2004). Competition STD NMR for the detection of high-affinity ligands and NMR-based screening. Magn. Reson. Chem..

[101] Schmitz U., James T.L. (1995). How to generate accurate solution structures of double-helical nucleic acid fragments using nuclear magnetic resonance and restrained molecular dynamics. Methods Enzymol..

[102] Briinger A.T., Adams P.D., Clore G.M. (1998). Crystallography & NMR system: A new software suite for macromolecular structure determination. Acta Crystallogr. D Biol. Crystallogr..

[103] Hirao I., Kawai G., Yoshizawa S. (1994). Most compact hairpin-turn structure exerted by a short DNA fragment, d(GCGAAGC) in solution: An extraordinarilystable structure resistant to nucleases and heat. Nucleic Acids Res..

